# Estrogen Enhances Endothelial Differentiation and Angiogenic Function of Adipose-Derived Stromal Cells to Improve Therapeutic Outcomes in Critical Limb Ischemia

**DOI:** 10.3390/cells15100885

**Published:** 2026-05-12

**Authors:** Hsin-Ju Chiang, Chang-Chun Hsiao, Steve Leu

**Affiliations:** 1 Graduate Institute of Clinical Medical Sciences, College of Medicine, Chang Gung University, Taoyuan 333323, Taiwan; haubay@gmail.com; 2 AN-AN Women and Children Clinic, Kaohsiung 807038, Taiwan; 3 Division of Pulmonary and Critical Care Medicine, Kaohsiung Chang Gung Memorial Hospital and Chang Gung University College of Medicine, Kaohsiung 833253, Taiwan; 4 Institute for Translational Research in Biomedicine, Kaohsiung Chang Gung Memorial Hospital, Kaohsiung 833253, Taiwan; 5 Department of Biotechnology, College of Life Science, Kaohsiung Medical University, Kaohsiung 807378, Taiwan; 6 Department of Nutrition and Health Science, Fooyin University, Kaohsiung 831301, Taiwan

**Keywords:** adipose-derived stromal cells, estrogen, endothelial differentiation, critical limb ischemia

## Abstract

Background: Aging, especially after menopause, reduces the quantity and function of adult stem cells. Estrogen deficiency impairs proliferation, differentiation, and regenerative capacity. This study evaluated whether estrogen enhances endothelial differentiation of adipose-derived stromal cells (ADSCs) and improves therapeutic efficacy in critical limb ischemia (CLI). Methods: Male-derived ADSCs were assessed in vitro for endothelial differentiation using flow cytometry, biochemical assays, and angiogenesis analyses. Therapeutic effects were tested in a rat CLI model using endothelial-differentiated ADSCs (ED-ADSCs) with or without 17β-estradiol (E2). An ovariectomized (OVX) model examined estrogen deficiency and supplementation in vivo. Results: E2 promoted endothelial differentiation, increasing ERα/ERβ expression and activating PI3K/Akt/eNOS and MAPK signaling. This led to elevated VEGF expression, enhanced tube formation, and increased CD34^+^, KDR^+^, and CD31^+^ cell populations. In vivo, E2-pretreated ED-ADSCs significantly improved blood flow recovery. Estrogen deficiency reduced endothelial progenitor populations, which were restored by E2 supplementation. Conclusions: Estrogen modulates endothelial-associated characteristics and angiogenesis-related responses of ADSCs via ER-associated signaling, and may contribute to improved functional outcomes in ischemic conditions. E2 preconditioning may represent a potential strategy for stem cell-based therapy in estrogen-deficient settings.

## 1. Introduction

Mesenchymal stromal cells (MSCs), historically referred to as mesenchymal stem cells, are multipotent cells that can be isolated from various tissues, including adipose tissue, bone marrow, placenta, umbilical cord, and peripheral blood [1,2]. Owing to their multilineage differentiation potential and immunomodulatory properties, MSCs have been widely investigated in regenerative medicine for inflammatory and ischemia-related diseases [3]. Among MSCs from different sources, adipose-derived mesenchymal stromal cells (ADMSCs; hereafter referred to as ADSCs) offer practical advantages, including abundant availability, minimally invasive harvesting, and favorable safety profiles [4,5,6]. In addition, ADSCs have been widely recognized to possess broad therapeutic properties, including tissue regenerative capacity, antioxidant activity, anti-apoptotic effects, and immunomodulatory functions [5,6,7,8]. However, although ADSCs exhibit pro-angiogenic effects primarily through paracrine mechanisms, their intrinsic endothelial differentiation capacity remains relatively limited, which may partially constrain their therapeutic efficacy in ischemic injury [9,10], particularly under conditions associated with impaired vascular repair, such as the postmenopausal state [11,12].

Postmenopausal women have an increased risk of numerous diseases, including cardiovascular disease (CVD), osteoporosis, vasomotor symptoms, and urogenital disorders [13]. Among these conditions, CVD remains the leading cause of mortality in women over 50 years of age [14]. Notably, postmenopausal women exhibit poorer outcomes following major cardiovascular events, partly due to accelerated endothelial dysfunction, which also contributes to a higher incidence and worse prognosis of peripheral arterial disease [15,16,17]. The decline in circulating estrogen levels has therefore led to the widespread use of hormone replacement therapy (HRT) [18]. Accumulating evidence indicates that estrogen increases endothelial progenitor cell numbers and promotes endothelial repair [19,20,21]. However, long-term systemic estrogen-based HRT is also linked to increased risks of hormone-related malignancies, which limits its broad clinical application [22,23].

In this context, estrogen-based modulation of ADSCs may represent a promising strategy to enhance endothelial differentiation and pro-angiogenic capacity while avoiding the adverse effects of prolonged systemic estrogen exposure. Estrogen receptor (ER) signaling activates multiple downstream pathways, including PI3K/Akt and MAPK cascades, in a context-dependent manner. In particular, activation of the PI3K/Akt/eNOS axis is well recognized to play a central role in endothelial function and angiogenesis [24,25]. Notably, accumulating evidence indicates that different MAPK pathways exert distinct functional roles in endothelial and progenitor cells, with ERK primarily associated with cell migration, proliferation, and survival [26,27], whereas JNK and p38 are more closely linked to stress-responsive signaling, angiogenic activation, and cytoskeletal remodeling [28,29]. Accordingly, we hypothesized that E2 may enhance endothelial differentiation of ADSCs through activation of the PI3K/Akt/eNOS pathway and differential engagement of MAPK signaling cascades.

To test this hypothesis, we evaluated the effects of E2 on ADSC differentiation and angiogenic function in vitro and further assessed the therapeutic efficacy of E2-pretreated endothelial-differentiated ADSCs (ED-ADSCs) in a rat model of critical limb ischemia. Male-derived ADSCs were used in vitro to minimize interference from endogenous estrogen and to better delineate cell-intrinsic estrogen signaling. In parallel, an ovariectomized (OVX) female rat model with E2 supplementation was employed to validate the physiological relevance of estrogen-dependent effects in vivo.

## 2. Materials and Methods

### 2.1. Ethics

All animal experimental procedures were approved by the Institutional Animal Care and Use Committee of Kaohsiung Chang Gung Memorial Hospital (Approval No. 2017050401) and were conducted in accordance with the National Institutes of Health Guide for the Care and Use of Laboratory Animals. All methods are reported in accordance with the ARRIVE guidelines (https://arriveguidelines.org) for the reporting of animal experiments [30].

### 2.2. Animals and Experimental Groups

Animal experiments in this study were conducted in an Association for Assessment and Accreditation of Laboratory Animal Care International (AAALAC)-certified animal facility in our hospital under controlled temperature and light conditions (24 °C; 12-h light/12-h dark cycle). At the designated experimental endpoint, animals were deeply anesthetized with inhalational isoflurane and euthanized by isoflurane overdose followed by exsanguination. Death was confirmed by the absence of spontaneous respiration, heartbeat, and reflex responses prior to tissue harvesting. Hindlimb skeletal muscle (quadriceps) and femurs were then immediately collected for subsequent immunofluorescent staining or isolation of bone marrow cells.

To investigate the in vitro effects of estrogen on endothelial differentiation of adipose-derived stromal cells (ADSCs), and to avoid interference from endogenous estrogen, abdominal adipose tissue was harvested in advance from eight-week-old male Sprague-Dawley (SD) rats (weighing 250–300 g; Charles River Technology, BioLASCO Co., Ltd., Taipei, Taiwan) for ADSC isolation and culture. After 14 days of endothelial differentiation, cells were used for subsequent transplantation in the CLI model. A total of 24 rats were randomly assigned to four groups (*n* = 6 per group): (1) sham control (SC); (2) CLI; (3) CLI + ED-ADSC; and (4) CLI + E2-ED-ADSC, in which ED-ADSCs were pretreated with 17β-estradiol (E2). In the treatment groups, cells were administered via intramuscular injection.

To assess the effects of in vivo estrogen supplementation on endothelial differentiation of ADSCs, eighteen twelve-week-old female Sprague-Dawley (SD) rats (weighing 180–220 g; Charles River Technology, BioLASCO Co., Ltd., Taipei, Taiwan) were randomly assigned to three groups (*n* = 6 per group): (1) sham-operated control (SC), (2) ovariectomized (OVX), and (3) ovariectomized with estrogen supplementation (OVX + E2). Ovariectomy was performed at 12 weeks of age. At 18 weeks of age, rats in the OVX + E2 group received 17β-estradiol (E2; 200 μg/kg/day; Sigma-Aldrich) via intraperitoneal injection for 14 consecutive days. Serum E2 levels were measured prior to sacrifice to confirm the effectiveness of estrogen supplementation. At 24 weeks of age, animals were euthanized, and bone marrow and abdominal adipose tissues were harvested for subsequent cell isolation and analyses.

### 2.3. Ovariectomy

Bilateral ovariectomy (OVX) was performed under general anesthesia using inhalational isoflurane. Rats were anesthetized with isoflurane (induction at 5% and maintenance at 2% in oxygen) and placed in the prone position on a temperature-controlled heating pad to maintain body temperature at approximately 37 °C throughout the procedure. After shaving the dorsal surgical area, the skin was disinfected with povidone-iodine followed by 70% ethanol. A small bilateral dorsolateral skin incision (1.5 cm) was made caudal to the last rib. The underlying muscle layer was bluntly dissected to expose the peritoneal cavity. The ovary and surrounding fat pad were gently exteriorized, and the oviduct was ligated with absorbable sutures before excision of the ovary. The uterine horn was then carefully returned to the abdominal cavity, and the same procedure was performed on the contralateral side. For sham-operated control (SC) animals, identical surgical procedures were carried out except that the ovaries were not removed. The muscle layer was closed with absorbable sutures, and the skin incision was closed using non-absorbable sutures. Animals were allowed to recover on a warming pad before being returned to their cages and monitored daily for signs of pain, infection, or wound dehiscence during the recovery period.

### 2.4. Isolation and Culture of Adipose-Derived Stromal Cells and Bone Marrow-Derived Cells

Cell isolation and culture procedures were performed as previously described [31,32]. with minor modifications. Briefly, abdominal adipose tissue was harvested under sterile conditions and minced into fragments smaller than 1 mm^3^ using sterile surgical scissors. The tissue was subsequently suspended in sterile saline and enzymatically digested with collagenase type I (Sigma-Aldrich, St. Louis, MO, USA) at a final concentration of 0.5 U/mL for 40 min at 37 °C with gentle agitation on an orbital shaker. Following digestion, the cell suspension was mechanically dissociated by gentle trituration using a 25 mL pipette for 2–3 min and centrifuged at 600× *g* for 5 min at room temperature. The floating adipose layer and supernatant were carefully removed, and the cell pellet was washed with 40 mL sterile saline and centrifuged again under the same conditions. The resulting cell suspension was sequentially filtered through 100 μm and 40 μm cell strainers (BD Falcon, BD Biosciences, San Jose, CA, USA) to obtain a stromal vascular fraction-enriched cell population. Isolated adipose-derived stromal cells (ADSCs) were cultured in endothelial growth medium (EGM-2; Lonza, Walkersville, MD, USA) supplemented with the EGM-2 BulletKit to induce endothelial differentiation. Cells were treated with or without E2 treatment (17β-estradiol; 100 nM, Sigma-Aldrich). E2 was dissolved in ethanol, and an equivalent concentration of vehicle was added to control cultures to exclude solvent-related effects. For bone marrow-derived cells (BMCs), bone marrow was aseptically flushed from the distal femurs of each animal. Bone marrow mononuclear cells (BM-MNCs) were isolated by Ficoll-Paque (Amersham) density-gradient centrifugation and subsequently cultured in EGM-2 medium under endothelial-promoting conditions. Cells were maintained under adherent culture conditions throughout the 14-day endothelial differentiation period and were subsequently used for further analyses.

### 2.5. Tube Formation Assay

For the in vitro angiogenesis assay, 96-well plates (Nunc, Waltham, MA, USA) were coated with growth factor-reduced Matrigel (40 μL per well; BD Biosciences, Piscataway, NJ, USA) and allowed to polymerize at 37 °C for 30 min. A total of 3 × 10^4^ cells were seeded onto the Matrigel-coated wells in EGM-2 medium containing standard endothelial growth supplements. Tube formation was observed and imaged 6 h after seeding using an IX51 inverted microscope (Olympus, Tokyo, Japan). Tube formation was quantified by assessing the number of tube-like structures, branching points, and total tube length using ImageJ 1.52q software (National Institutes of Health, Bethesda, MD, USA). Experiments were performed using cells derived from independent animals, with each condition tested in triplicate wells.

### 2.6. Transwell Migratory Assay

Transwell membranes (5 μm pore size; Costar, Corning Inc., Corning, NY, USA) were coated on both sides with fibronectin (2.5 μg/mL; Roche, Mannheim, Germany) overnight at 4 °C. A total of 5 × 10^4^ cells were resuspended in EGM-2 medium containing standard endothelial growth supplements and 0.5% fetal bovine serum (FBS), and seeded into the upper chamber. The lower chamber was filled with EGM-2 medium supplemented with 10% FBS to establish a chemotactic gradient. The transwell system was incubated at 37 °C in a humidified atmosphere with 5% CO_2_ for 18 h. After incubation, non-migrated cells on the upper surface of the membrane were gently removed using a cotton swab. Cells that had migrated to the lower surface were fixed with 4% paraformaldehyde and stained with 4′,6-diamidino-2-phenylindole (DAPI) for nuclear visualization. Migrated cells were quantified by counting DAPI-positive nuclei in five randomly selected high-power fields per membrane using a fluorescence microscope (Olympus, Tokyo, Japan). Image analysis was performed using Image-Pro Plus 6.0 software (Media Cybernetics, Rockville, MD, USA). Each experiment was performed in triplicate. Fibronectin coating was applied specifically for the migration assay, and ED-ADSCs were not routinely cultured on fibronectin-coated surfaces under standard conditions.

### 2.7. Quantitative Reverse Transcription-Polymerase Chain Reaction

Total RNA was extracted from ADSCs using the RNeasy Mini Kit (Qiagen, Hilden, Germany) according to the manufacturer’s instructions. The quantity and purity of RNA were assessed prior to downstream analysis. Reverse transcription was performed using 10 ng of total RNA with TaqMan^®^ Universal PCR Master Mix (no AmpErase UNG; Applied Biosystems, Foster City, CA, USA) and gene-specific TaqMan^®^ probes. Quantitative real-time PCR (qRT-PCR) was conducted using the Applied Biosystems 7900HT Sequence Detection System (Applied Biosystems). Each reaction was performed in a total volume of 20 μL according to the manufacturer’s protocol. The thermal cycling conditions were as follows: initial denaturation at 95 °C for 10 min, followed by 40 cycles of 95 °C for 15 s and 60 °C for 60 s. All samples were analyzed in triplicate. Relative mRNA expression levels were calculated using the comparative cycle threshold (Ct) method (2^−ΔΔCt^), with β-actin serving as the internal control for normalization.

### 2.8. Western Blot

Equal amounts of protein extracts (10–30 μg) from ADSCs cultured under endothelial differentiation conditions in EGM-2 medium supplemented with growth factors, with or without E2, were separated by SDS-PAGE using 7.5–12% gradient polyacrylamide gels. No serum starvation or acute stimulation was applied prior to protein collection. Following electrophoresis, proteins were electrotransferred onto polyvinylidene difluoride (PVDF) membranes (Amersham Biosciences, Buckinghamshire, UK). Membranes were blocked with 5% nonfat dry milk in Tris-buffered saline containing 0.05% Tween-20 (TBS-T) for 30 min at room temperature to prevent nonspecific binding. The membranes were then incubated with primary antibodies against phosphorylated Akt (1:1000, Cell Signaling Technology, Danvers, MA, USA), total Akt (1:1000, Cell Signaling Technology, Danvers, MA, USA), phosphorylated ERK (1:1000, Cell Signaling Technology, Danvers, MA, USA), total ERK (1:1000, Cell Signaling Technology, Danvers, MA, USA), phosphorylated JNK (1:1000, Abcam, Cambridge, UK), total JNK (1:1000, Abcam, Cambridge, UK), phosphorylated p38 MAPK (1:1000, Sigma-Aldrich, St. Louis, MO, USA), total p38 MAPK (1:5000, Abcam, Cambridge, UK), β-actin (1:1000, Cell Signaling Technology, Danvers, MA, USA), VEGF (1:1000, Cell Signaling Technology, Danvers, MA, USA), and phosphorylated eNOS (1:500, Abcam, Cambridge, UK) for 1 h at room temperature. After washing with TBS-T, membranes were incubated with horseradish peroxidase (HRP)-conjugated goat anti-mouse or goat anti-rabbit secondary antibodies (1:1000, Cell Signaling Technology, Danvers, MA, USA) for 1 h at room temperature. Protein signals were detected using an enhanced chemiluminescence (ECL) detection system (PerkinElmer, Waltham, MA, USA). Band intensities were quantified using ImageJ software (National Institutes of Health, Bethesda, MD, USA). Phosphorylated protein levels were normalized to their corresponding total protein levels, and all protein expression levels were further normalized to β-actin. For Western blot analysis, *n* = 6 represents six independent biological samples, each analyzed as an individual lane. For PI3K analysis, the ~85 kDa band corresponding to the p85 regulatory subunit was used for quantification.

### 2.9. Induction of Critical Limb Ischemia and Cell Transplantation

To investigate the therapeutic effects of endothelial-differentiated adipose-derived stromal cells (ED-ADSCs) on critical limb ischemia (CLI), abdominal adipose tissue was first harvested from eight-week-old male Sprague-Dawley (SD) rats for ADSC isolation and culture. After 14 days of endothelial differentiation, ED-ADSCs, with or without 17β-estradiol (E2) pretreatment, were prepared for transplantation. CLI was then induced in the left hindlimb as previously described with minor modifications. Briefly, rats were anesthetized with inhalational isoflurane, and the left femoral artery was surgically exposed through a longitudinal inguinal incision under sterile conditions. The femoral artery and its major branches were ligated, and the intervening vascular segment was excised to ensure complete interruption of blood flow. Successful induction of CLI was confirmed by gross evidence of pallor and reduced perfusion in the affected limb. At 30 min after CLI induction, rats received intramuscular transplantation of ED-ADSCs or E2-pretreated ED-ADSCs into the ischemic hindlimb muscles. A total of 1 × 10^6^ cells in 100 μL sterile saline, a dose within the range reported in rodent models of hindlimb ischemia [32,33], was injected into four sites within the ischemic region using a sterile syringe. Animals in the control CLI group received an equal volume of vehicle. To evaluate in vivo retention of transplanted cells, additional animals in the ED-ADSC and E2-ED-ADSC groups received DiR- or DiI-labeled cells before transplantation for subsequent cell-tracking analysis.

### 2.10. Measurement of Hindlimb Blood Perfusion by Laser Doppler Imaging

Blood flow measurement was performed using a Laser Doppler system as described in our previous studies [34]. Briefly, limb perfusion in both normal and ischemic hindlimbs was assessed under inhalational anesthesia with 2.0% isoflurane at baseline (prior to CLI induction, day 0) and on days 3, 7, and 14 following CLI surgery. For each measurement, animals were placed in a supine position on a temperature-controlled warming pad maintained at 37 °C. Both hindlimbs were carefully shaved to minimize signal interference. Blood perfusion was then quantified using a Laser Doppler scanner (moorLDLS, Moor Instruments Ltd., Devon, UK). The acquired perfusion data were recorded and analyzed using the manufacturer’s software. After the final measurement on day 14, animals were euthanized, and quadriceps muscle tissues were harvested for immunofluorescent staining.

### 2.11. Immunofluorescent Staining

For immunofluorescence staining, isolated quadriceps muscles were embedded in optimal cutting temperature (OCT) compound and cryosectioned. Tissue sections were fixed with 4% paraformaldehyde and permeabilized with 0.5% Triton X-100 in phosphate-buffered saline (PBS). After blocking with appropriate blocking buffer, sections were incubated overnight at 4 °C with primary antibodies against stem cell antigen-1 (Sca-1; Abcam, Cambridge, UK). Following washing with PBS, sections were incubated with Alexa Fluor 488-conjugated secondary antibodies (Invitrogen, Carlsbad, CA, USA) at room temperature for 1 h. Nuclei were counterstained with 4′,6-diamidino-2-phenylindole (DAPI). Fluorescence images were acquired using a fluorescence microscope.

### 2.12. Statistical Analysis

Data are presented as mean ± standard deviation (SD). Comparisons between two groups were performed using the unpaired Student’s *t*-test. Differences among multiple groups were analyzed using one-way analysis of variance (ANOVA), followed by Tukey’s multiple comparisons post hoc test. Statistical analyses were performed using Prism 11 software (GraphPad Software, La Jolla, CA, USA). A *p* value < 0.05 was considered statistically significant.

## 3. Results

### 3.1. Estrogen Treatment Increases Estrogen Receptor Expression in Adipose-Derived Stromal Cells

Prior to evaluating the effects of estrogen on endothelial differentiation of adipose-derived stromal cells (ADSCs), we first examined the expression of estrogen receptors (ERα and ERβ) in ADSCs undergoing endothelial differentiation (ED-ADSCs). To minimize potential sex-related variability and facilitate broader translational applications, ADSCs were isolated from the abdominal adipose tissue of 8-week-old male rats. The cells were cultured in endothelial growth medium (EGM-2) for 14 days to induce endothelial differentiation in the presence or absence of estrogen treatment with 17β-estradiol (E2).

**Figure 1:**
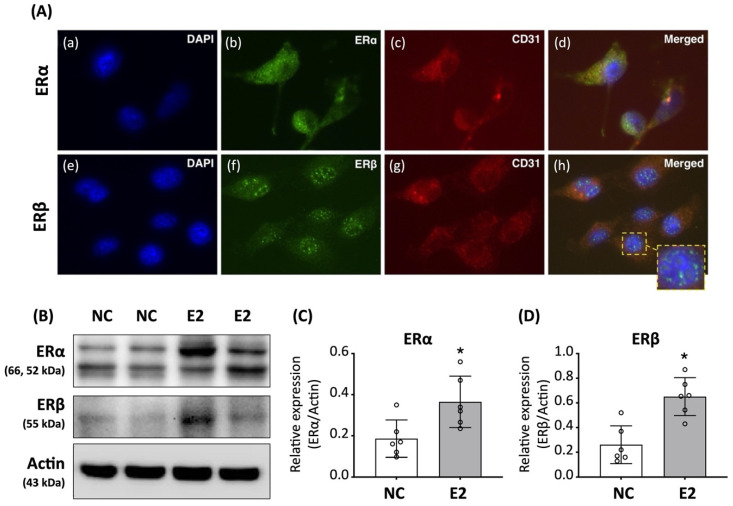
Expression and subcellular distribution of estrogen receptors in endothelial-differentiated adipose-derived stromal cells (ED-ADSCs). Adipose-derived stromal cells (ADSCs) were isolated from abdominal adipose tissue of 8-week-old male rats and cultured in EGM-2 medium for 14 days to induce endothelial differentiation in the presence or absence of 17β-estradiol (E2). (**A**) Immunofluorescence staining showing the expression and subcellular localization of estrogen receptor (ER) α and β in ED-ADSCs. (**B**) Representative Western blot images of ERα and ERβ expression in ED-ADSCs with or without E2 treatment. (**C**) Quantification of ERα protein levels. (**D**) Quantification of ERβ protein levels. NC, normal control; E2, 17β-estradiol. Data are presented as mean ± SD (*n* = 6 per group). * *p* < 0.05 versus NC.

Immunofluorescence staining revealed distinct subcellular localization patterns of ERα and ERβ in ED-ADSCs. In E2-treated cells, ERα was predominantly distributed in the cytoplasm, whereas ERβ was mainly localized in the nucleus with a speckled pattern (Figure 1A). Western blot analysis further demonstrated that E2 treatment significantly increased the expression levels of both ERα and ERβ in ED-ADSCs compared with untreated controls (Figure 1B,C).

### 3.2. Estrogen Promotes Endothelial Differentiation and Enhances Angiogenic Activity of Adipose-Derived Mesenchymal Stem Cells

**Figure 2:**
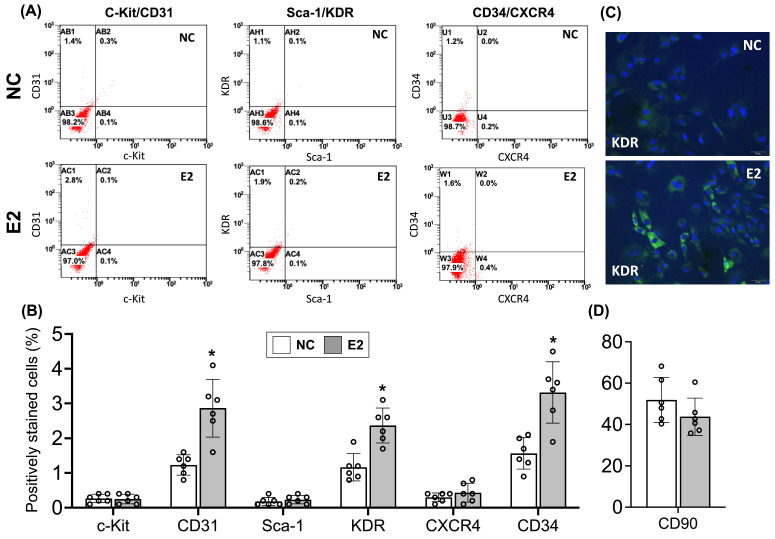
Flow cytometric and immunofluorescence analyses of endothelial differentiation markers in ED-ADSCs with E2 treatment. Flow cytometric analysis was performed to evaluate the expression of surface markers associated with endothelial differentiation (CD31 and KDR/VEGFR2), progenitor characteristics (CD34), and stemness or migratory capacity (c-Kit, Sca-1, CXCR4, and CD90) in endothelial-differentiated adipose-derived stromal cells (ED-ADSCs) with or without 17β-estradiol (E2) treatment. (**A**) Representative flow cytometry dot plots showing marker expression profiles. (**B**) Quantification of marker-positive cell populations. (**C**) Immunofluorescence staining showing KDR expression in ED-ADSCs. (**D**) Quantification of CD90^+^ cell populations. NC, normal control; E2, 17β-estradiol. Data are presented as mean ± SD (*n* = 6 per group). * *p* < 0.05 versus NC.

Following the observed upregulation of estrogen receptors in ED-ADSCs, we next investigated whether E2 enhances endothelial differentiation and angiogenic function of ADSCs. Flow cytometric analysis was performed using antibodies against markers associated with endothelial differentiation (CD31 and KDR/VEGFR2), progenitor characteristics (CD34), and stemness or migratory capacity (c-Kit, CD90, and CXCR4). Compared with untreated controls, E2 treatment significantly increased the proportions of CD31^+^ and KDR^+^ cells in ED-ADSCs (Figure 2A,B), indicating enhanced endothelial differentiation. In addition, the percentage of CD34^+^ cells, a marker of hematopoietic and progenitor populations, was also elevated following E2 treatment, suggesting an expansion of cells with angiogenic potential. Immunofluorescence staining further confirmed the upregulation of KDR expression in E2-treated ED-ADSCs (Figure 2C), supporting the promotive effect of E2 on endothelial commitment.

In contrast, the proportions of CXCR4^+^ and c-Kit^+^ cells (Figure 2A,B), as well as CD90^+^ cells (Figure 2D), were not significantly altered by E2 treatment. Given that CXCR4 is primarily associated with cell migration and homing, c-Kit reflects progenitor/stem cell characteristics, and CD90 is a canonical mesenchymal stromal cell marker, these findings suggest that E2 preferentially promotes endothelial commitment without markedly affecting migratory signaling pathways or the core mesenchymal identity of ADSCs.

### 3.3. E2 Modulates Angiogenesis-Associated Properties in Endothelial-Differentiated ADSCs

**Figure 3:**
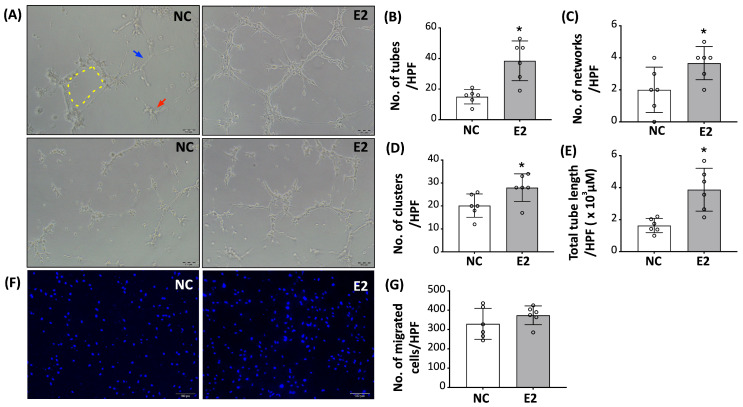
Effects of E2 on angiogenesis-associated and migratory properties of ED-ADSCs. A Matrigel-based in vitro tube formation assay and a transwell migration assay were performed to evaluate angiogenesis-associated activity and migratory properties of endothelial-differentiated adipose-derived stromal cells (ED-ADSCs) with or without 17β-estradiol (E2) treatment. (**A**) Representative images of tube formation under control (NC) and E2-treated conditions. Images from different fields of view at the same magnification are shown to illustrate the overall pattern of network formation. The yellow dashed box indicates network formation, blue arrows indicate tubes, and red arrows indicate clusters. (**B**–**E**) Quantitative analysis of tube formation, including the number of tubes (**B**), networks (**C**), clusters (**D**), and total tube length (**E**) per high-power field (HPF). (**F**) Representative images of migrated cells in a transwell migration assay. (**G**) Quantification of migrated cells per HPF. NC, normal control; E2, 17β-estradiol. Data are presented as mean ± SD (*n* = 6 per group). * *p* < 0.05 versus NC.

Following the changes in endothelial-associated characteristics observed in E2-treated ED-ADSCs, we next examined whether these phenotypic changes were associated with functional properties. A Matrigel-based in vitro tube formation assay was performed to assess tube formation behavior. Compared with untreated controls, E2-treated ED-ADSCs showed increased formation of capillary-like structures (Figure 3A). Quantitative analysis demonstrated that E2 treatment increased the number of tubes, networks, and clusters, as well as total tube length (Figure 3B–E).

To further evaluate the effect of E2 on cell motility, a transwell migration assay was conducted. No statistically significant difference in migratory capacity was observed between E2-treated and control ED-ADSCs (Figure 3F,G).

### 3.4. Estrogen Treatment Modulates PI3K/Akt/eNOS Signaling in ADSCs

To explore the underlying signaling mechanisms, we examined whether E2 modulates the PI3K/Akt/eNOS pathway in ED-ADSCs. Estrogen receptor-mediated PI3K/Akt signaling has been implicated in eNOS activation and endothelial-associated responses.

**Figure 4:**
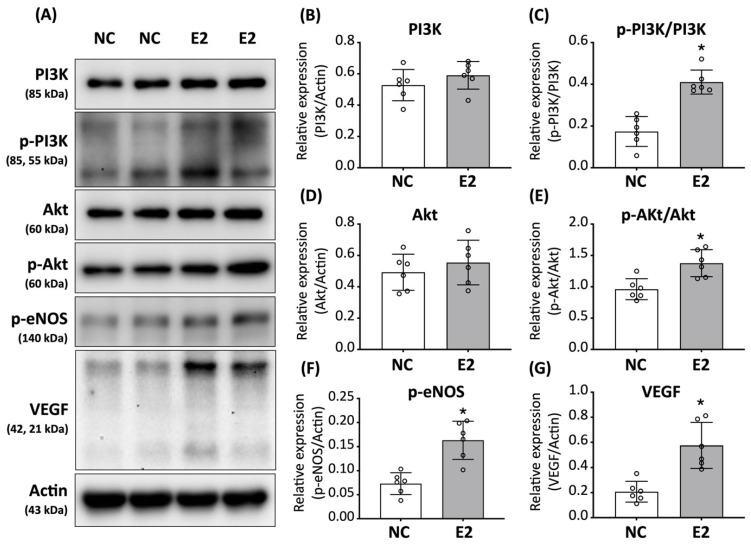
Effects of E2 on PI3K/Akt/eNOS signaling and VEGF expression in ED-ADSCs. Western blot analysis was performed to evaluate the expression and phosphorylation levels of signaling molecules in endothelial-differentiated adipose-derived stromal cells (ED-ADSCs) with or without 17β-estradiol (E2) treatment. (**A**) Representative Western blot images of PI3K, phosphorylated PI3K (p-PI3K), Akt, phosphorylated Akt (p-Akt), phosphorylated eNOS (p-eNOS), VEGF, and β-actin. (**B**–**G**) Quantitative analysis of total protein expression and phosphorylation levels of PI3K, Akt, eNOS, and VEGF. NC, normal control; E2, 17β-estradiol. Data are presented as mean ± SD (*n* = 6 per group). * *p* < 0.05 versus NC.

As shown in Figure 4A, E2 treatment did not significantly alter the total protein expression levels of PI3K and Akt (Figure 4B,D). However, the phosphorylation levels of PI3K and Akt were modestly increased in E2-treated ED-ADSCs compared with untreated controls (Figure 4C,E). Consistently, phosphorylated eNOS levels were also increased following E2 treatment (Figure 4F). In addition, VEGF expression showed an increasing trend in E2-treated ED-ADSCs (Figure 4G). These findings suggest that E2 modulates PI3K/Akt/eNOS signaling under endothelial differentiation conditions, consistent with a modest enhancement of endothelial-associated signaling rather than robust activation.

### 3.5. E2 Selectively Modulates JNK and p38 MAPK Signaling in ED-ADSCs

We next examined whether MAPK signaling pathways are involved in E2-mediated responses in ED-ADSCs. MAPK pathways, including ERK, JNK, and p38, are known to participate in estrogen signaling and regulation of cellular function.

**Figure 5:**
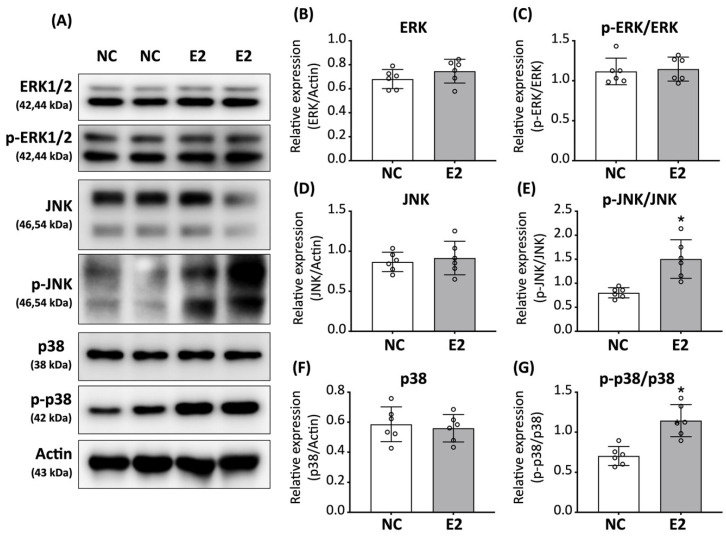
Effects of E2 on MAPK signaling pathways in ED-ADSCs. Western blot analysis was performed to evaluate the expression and phosphorylation levels of MAPK signaling proteins in endothelial-differentiated adipose-derived stromal cells (ED-ADSCs) with or without 17β-estradiol (E2) treatment. (**A**) Representative Western blot images of ERK1/2, phosphorylated ERK1/2 (p-ERK1/2), JNK, phosphorylated JNK (p-JNK), p38, phosphorylated p38 (p-p38), and β-actin. (**B**–**G**) Quantitative analysis of total protein expression and phosphorylation levels of ERK1/2, JNK, and p38. NC, normal control; E2, 17β-estradiol. Data are presented as mean ± SD (*n* = 6 per group). * *p* < 0.05 versus NC.

As shown in Figure 5A, E2 treatment did not significantly alter ERK1/2 expression or phosphorylation (Figure 5B,C). Similarly, total protein levels of JNK and p38 remained unchanged between groups (Figure 5D,F). However, phosphorylation levels of JNK and p38 were modestly increased in E2-treated ED-ADSCs (Figure 5E,G). These results suggest that E2 selectively modulates JNK and p38 signaling pathways under endothelial differentiation conditions. This pattern is consistent with a context-dependent modulation of cellular responses rather than strong activation of MAPK signaling.

### 3.6. Estrogen Treatment Modulates mRNA Expression of Angiogenesis-Associated Genes

In addition to extra-nuclear signaling, activated estrogen receptors are known to regulate gene transcription through direct DNA binding and recruitment of transcriptional co-regulators. Accordingly, we investigated whether estrogen modulates angiogenesis-related gene expression at the transcriptional level.

**Figure 6:**
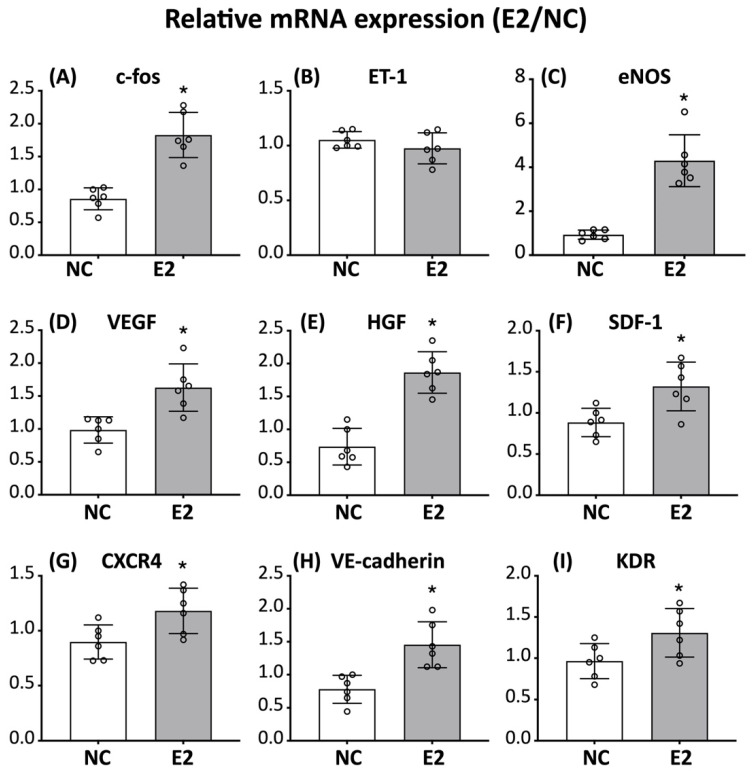
Effects of E2 on mRNA expression of angiogenesis-associated genes in ED-ADSCs. Quantitative analysis of mRNA expression was performed to assess the expression of angiogenesis-associated genes in endothelial-differentiated adipose-derived stromal cells (ED-ADSCs) with or without 17β-estradiol (E2) treatment. (**A**–**I**) Relative mRNA expression levels of c-fos, ET-1, eNOS, VEGF, HGF, SDF-1, CXCR4, VE-cadherin, and KDR. NC, normal control; E2, 17β-estradiol. Data are presented as mean ± SD (*n* = 6 per group). * *p* < 0.05 versus NC.

Quantitative analysis of mRNA expression showed that E2 treatment increased the expression of c-fos, a transcription factor associated with estrogen-responsive signaling (Figure 6A). In contrast, the expression of endothelin-1 (ET-1) was not significantly altered (Figure 6B). E2 treatment was associated with increased mRNA levels of several angiogenesis-related genes, including eNOS, VEGF, HGF, and SDF-1 (Figure 6C–F). In addition, the expression of endothelial-associated markers, such as VE-cadherin and KDR, was moderately increased (Figure 6H,I), while CXCR4 showed a modest elevation (Figure 6G). Although most changes were modest and did not exceed a two-fold threshold, the overall pattern was consistent with E2-associated modulation of angiogenesis-related gene expression, with eNOS showing the most prominent increase.

### 3.7. E2 Enhances the Therapeutic Efficacy of ED-ADSCs in Promoting Blood Flow Recovery in Ischemic Limbs

To determine whether E2 treatment enhances the therapeutic efficacy of ED-ADSCs, we evaluated blood flow recovery in a rat model of critical limb ischemia (CLI) using laser Doppler perfusion imaging.

**Figure 7:**
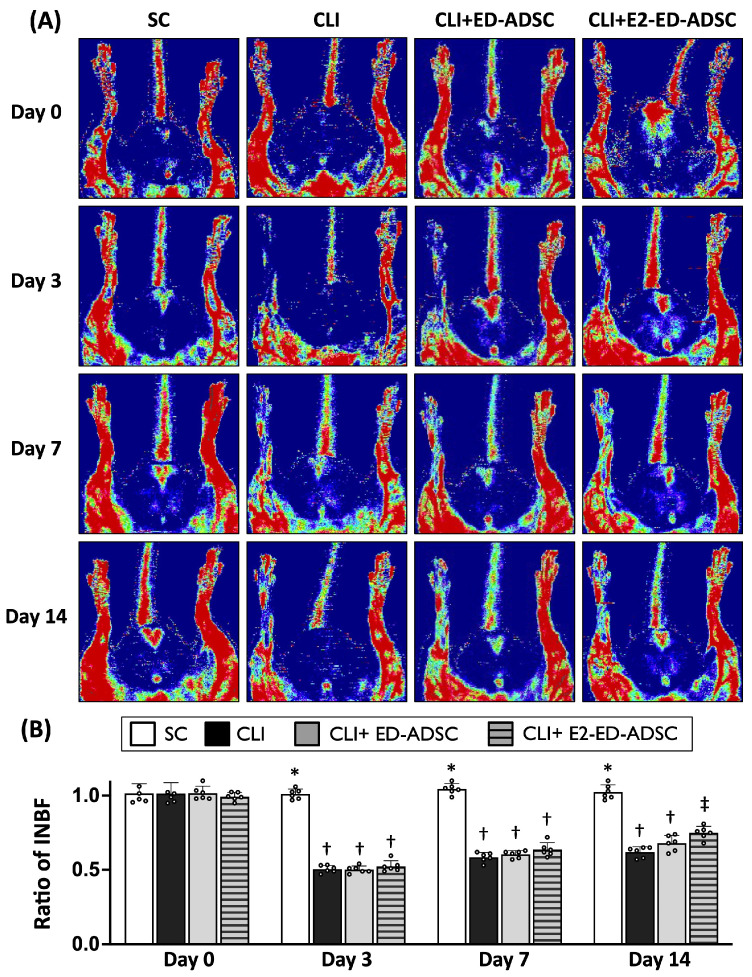
Blood flow recovery following ED-ADSC transplantation in a rat model of critical limb ischemia (CLI). Male Sprague-Dawley rats (8 weeks old) were used for adipose tissue harvest. Adipose-derived stromal cells (ADSCs) were cultured for 2 weeks and subjected to endothelial differentiation. Endothelial-differentiated ADSCs (ED-ADSCs), with or without E2 treatment, were administered via intramuscular injection 30 min after CLI induction. Blood flow recovery was evaluated at days 3, 7, and 14 using laser Doppler perfusion imaging. (**A**) Representative perfusion images of hindlimbs from each group at the indicated time points. (**B**) Quantitative analysis of blood flow recovery, expressed as the ratio of ischemic to non-ischemic limb blood flow (INBF). SC, sham control; CLI, critical limb ischemia; E2, 17β-estradiol; INBF, ischemic to non-ischemic limb blood flow. Data are presented as mean ± SD (*n* = 6 per group). Statistical differences among multiple groups were analyzed using one-way ANOVA followed by Tukey’s multiple comparisons post hoc test. Groups with different symbols (*, †, ‡) indicate statistically significant differences (*p* < 0.05), whereas groups sharing the same symbol are not significantly different.

As shown in Figure 7A, no significant differences in limb perfusion were observed among groups at baseline (day 0). At day 3 after CLI induction, all ischemic groups exhibited a marked reduction in perfusion compared with the sham control group, with no significant differences among treatment groups. By days 7 and 14 post-CLI, rats treated with ED-ADSCs showed a trend toward improved blood flow recovery compared with the CLI group, although the difference did not reach statistical significance. In contrast, E2-treated ED-ADSCs resulted in significantly greater blood flow recovery, particularly at day 14 (Figure 7B). These findings indicate that E2 enhances the therapeutic efficacy of ED-ADSCs in promoting blood flow recovery in ischemic limbs.

### 3.8. Transplanted ED-ADSCs Persist in Ischemic Tissue and Exhibit Progenitor-Associated Features

**Figure 8:**
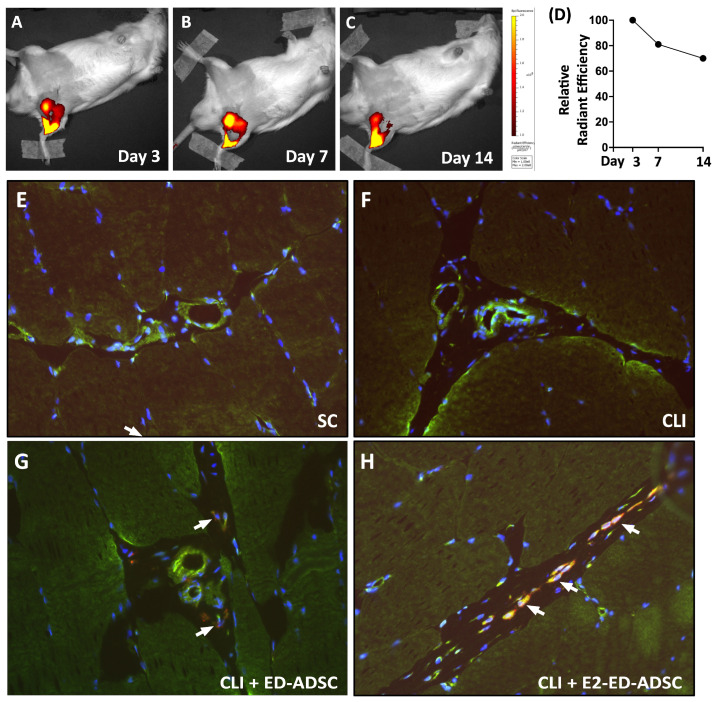
In vivo tracking and phenotypic characterization of transplanted ED-ADSCs in ischemic tissue. (**A**–**C**) Representative in vivo IVIS images showing the distribution of DiR-labeled ED-ADSCs at days 3, 7, and 14 after transplantation in a rat model of critical limb ischemia (CLI). (**D**) Quantification of DiR signal intensity, expressed as relative radiant efficiency normalized to day 3. (**E**–**H**) Immunofluorescence staining of ischemic quadriceps muscle at day 14. Transplanted ED-ADSCs were pre-labeled with DiI (red), and Sca-1 (green) was used as a progenitor cell marker. Nuclei were counterstained with DAPI (blue). DiI-positive cells were detected in ED-ADSC-treated groups. Co-localization of DiI and Sca-1 (arrows) was observed, with a higher number of DiI^+^/Sca-1^+^ cells in the E2-pretreated ED-ADSC group. These observations are qualitative and were not quantitatively assessed. SC, sham control; CLI, critical limb ischemia; ED-ADSC, endothelial-differentiated adipose-derived stromal cell; E2, 17β-estradiol.

To determine the in vivo retention and persistence of transplanted ED-ADSCs, longitudinal cell tracking was performed using DiR labeling for in vivo imaging system (IVIS) analysis, while DiI labeling was used for subsequent histological identification of transplanted cells (Figure 8).

As shown in Figure 8A–C, DiR signals were detected at the injection site at days 3, 7, and 14 after transplantation, indicating sustained localization of transplanted ED-ADSCs within ischemic tissue. Quantitative analysis normalized to the signal intensity at day 3 demonstrated a gradual decline over time; however, approximately 70% of the initial signal intensity was retained at day 14 (Figure 8D), indicating substantial persistence of transplanted cells.

To further confirm the presence and phenotype of transplanted cells at the tissue level, immunofluorescence staining was performed using DiI-labeled ED-ADSCs in combination with Sca-1, a progenitor cell marker (Figure 8E–H). DiI-positive cells were detected in ischemic quadriceps muscle at day 14, confirming the persistence of transplanted cells. Notably, co-localization of DiI and Sca-1 was observed. A higher number of DiI^+^/Sca-1^+^ cells appeared to be present in the E2-pretreated ED-ADSC group, suggesting enhanced progenitor-associated characteristics following E2 treatment. However, this observation was not quantitatively assessed and should be interpreted as a qualitative finding. Overall, these results primarily reflect the retention and localization of transplanted cells rather than direct evidence of vascularization.

### 3.9. Estrogen Supplementation Restores Endothelial Differentiation of Adipose- and Bone Marrow-Derived Cells in Ovariectomized Rats

**Figure 9:**
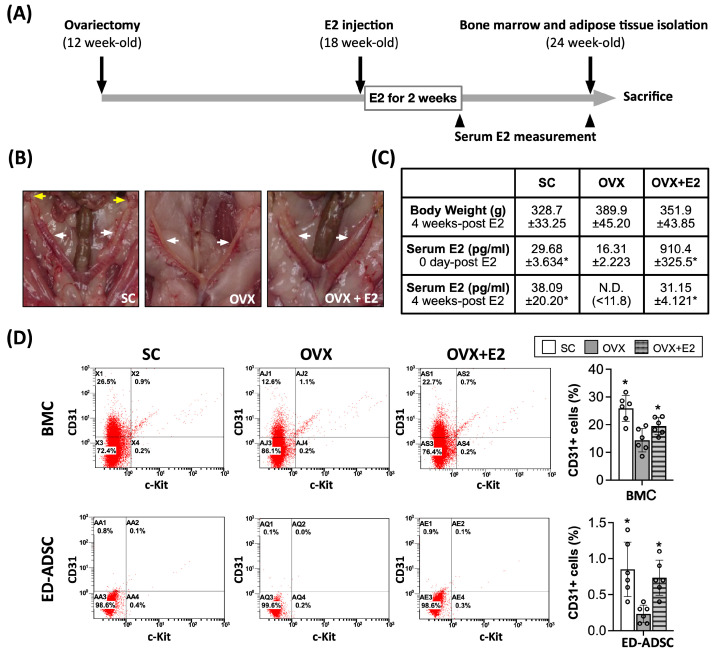
Effects of estrogen supplementation on endothelial differentiation capacity in an ovariectomized (OVX) rat model. (**A**) Schematic timeline of the OVX and estrogen (E2) supplementation protocol. (**B**) Representative images of reproductive tissues from sham control (SC), OVX, and OVX+E2 groups. Yellow arrows indicate ovaries, and white arrows indicate the uterus. (**C**) Body weight and serum E2 levels measured at the indicated time points. (**D**) Flow cytometric analysis of CD31^+^/c-Kit^+^ cell populations in bone marrow-derived cells (BMCs) and endothelial-differentiated adipose-derived stromal cells (ED-ADSCs). Representative dot plots and corresponding quantification are shown. CD31 was used as a representative endothelial-associated marker; therefore, these data reflect endothelial-associated changes rather than definitive endothelial differentiation or endothelial progenitor cell (EPC) identity. In contrast, c-Kit expression did not show significant changes across groups. SC, sham control; OVX, ovariectomy; E2, 17β-estradiol. Data are presented as mean ± SD (*n* = 6 per group). * *p* < 0.05 versus SC.

To further assess the physiological relevance of our findings, we examined whether estrogen supplementation influences endothelial-associated characteristics in an ovariectomized (OVX) rat model. Following ovariectomy and subsequent estrogen supplementation, mesenchymal stromal cells were isolated from bone marrow and adipose tissue and subjected to endothelial differentiation and flow cytometric analysis (Figure 9).

Gross examination revealed marked uterine atrophy in OVX rats, which was reversed by estrogen supplementation (Figure 9B), confirming the effectiveness of the ovariectomy model and estrogen replacement. Consistently, serum E2 levels were significantly reduced following ovariectomy and restored after estrogen supplementation (Figure 9C).

Flow cytometric analysis demonstrated a significant reduction in the proportion of CD31^+^ cells in endothelial-differentiated adipose-derived stromal cells (ED-ADSCs) from OVX rats (Figure 9D), suggesting reduced endothelial-associated characteristics under estrogen-deficient conditions. Estrogen supplementation partially restored the proportion of CD31^+^ cells in ED-ADSCs. A similar pattern was observed in bone marrow-derived cells, in which ovariectomy decreased, whereas estrogen supplementation increased, the percentage of CD31^+^ cells (Figure 9D), indicating that this effect is not restricted to adipose-derived cells. It should be noted that CD31 was used as a representative endothelial-associated marker in this analysis. Therefore, these findings should be interpreted as reflecting changes in endothelial-associated characteristics rather than definitive endothelial differentiation or endothelial progenitor cell (EPC) identity.

## 4. Discussion

The present study suggests that estrogen is involved in the regulation of endothelial-associated characteristics, functional responses, and therapeutic effects of adipose-derived stromal cells (ADSCs). We observed that estrogen receptor (ER) signaling is engaged in endothelial-differentiated ADSCs and is associated with changes in the expression of CD31, KDR, and angiogenesis-related genes. Functionally, E2 treatment was associated with modest enhancement of angiogenesis-related and migratory responses. This was accompanied by modulation of PI3K/Akt/eNOS and MAPK signaling pathways, as well as increased expression of VEGF and other pro-angiogenic factors.

In vivo, E2-preconditioned ED-ADSCs were associated with improved cell retention and maintenance of progenitor-like (Sca-1^+^) characteristics. These changes were accompanied by enhanced blood flow recovery in a rat model of critical limb ischemia. Conversely, estrogen deficiency induced by ovariectomy reduced endothelial-associated cell populations in both ADSCs and bone marrow-derived progenitor cells, which could be partially restored by estrogen supplementation. Collectively, these findings support a modulatory role of estrogen in coordinating endothelial-associated phenotypes, signaling responses, and regenerative function of mesenchymal stromal/progenitor cells.

The molecular mechanisms underlying the E2-mediated modulation of endothelial-associated characteristics in ED-ADSCs appear to involve coordinated engagement of multiple signaling pathways. Estrogen receptor (ER) signaling, including both ERα and ERβ, is known to participate in the regulation of endothelial function through genomic and non-genomic mechanisms [35,36,37]. In previous studies using PBMC-derived EPCs, ER isoforms were differentially regulated during endothelial maturation, with ERα expression increasing while ERβ progressively declined, suggesting a relatively restricted pattern of ER-associated signaling [38].

In contrast, our findings showed that exogenous E2 treatment in ED-ADSCs was associated with concurrent upregulation of both ERα and ERβ, along with distinct subcellular distribution patterns (Figure 1). This difference may suggest that E2 supplementation influences ER isoform dynamics and may broaden ER-associated signaling responses compared with classical EPC models. Consistent with this, E2 treatment was associated with modulation of downstream signaling pathways, including PI3K/Akt/eNOS and MAPK cascades, which have been implicated in endothelial-associated cellular responses [28,29,39].

In the present study, E2 increased the phosphorylation of JNK and p38, whereas ERK phosphorylation was not significantly altered. This selective modulation of MAPK pathways may reflect the context-dependent nature of estrogen signaling. Previous studies have demonstrated that ERK signaling plays a central role in VEGF-driven endothelial differentiation [40]. This suggests that ERK activity may already be sufficiently engaged under endothelial differentiation conditions in ED-ADSCs, thereby limiting further enhancement by E2 treatment. In addition, this pattern may also partially reflect a general stress-related response to E2 stimulation under these conditions. In contrast, JNK and p38 are more commonly associated with stress adaptation, cytoskeletal remodeling, and angiogenic responses [29,41,42], rather than with differentiation. Notably, these signaling patterns may also be influenced by the differential regulation of ER isoforms. ERβ has been reported to function as a modulatory counterpart to ERα and may preferentially influence non-classical or context-dependent signaling outputs [37]. Therefore, the concurrent upregulation of ERα and ERβ observed in this study may contribute to a shift toward selective activation of JNK and p38 rather than further enhancement of ERK signaling. Further studies are required to delineate the isoform-specific contributions of ER signaling and to clarify the mechanistic link between ER regulation and selective MAPK pathway activation in this context.

In addition, the upregulation of angiogenesis-related genes, including VEGF, SDF-1, and CXCR4, is consistent with previous studies demonstrating estrogen-mediated enhancement of pro-angiogenic transcriptional programs and cell mobilization [43,44,45]. The engagement of this broader and more integrated estrogen signaling network likely contributes to the endothelial-associated characteristics in E2-treated ED-ADSCs. Notably, the magnitude of changes in signaling and gene expression was modest, which may reflect the elevated baseline under endothelial differentiation conditions. Importantly, these effects were consistent across multiple readouts, including signaling activation, gene expression, and functional assays, supporting a modulatory role of E2 rather than robust induction of endothelial differentiation.

Although stem cell-based therapies for improving blood flow recovery in ischemic organ injury have gained increasing recognition [46,47], their therapeutic efficacy may be compromised under conditions of aging [11,12,48]. In particular, estrogen deficiency following menopause has been shown to impair the functional capacity of stem and progenitor cells, including reduced proliferative, migratory, and angiogenic abilities [38,49]. Such alterations may limit the effectiveness of cell-based therapies in clinical settings, especially in elderly and postmenopausal populations. Consistent with this concept, our ovariectomy (OVX) model showed a reduction in endothelial-associated cell populations in both adipose-derived stromal cells and bone marrow-derived progenitor cells, as evidenced by a decreased proportion of CD31^+^ cells (Figure 9). Estrogen supplementation was associated with partial restoration of endothelial-associated characteristics in both cell types, suggesting that estrogen contributes to the maintenance of progenitor cell function across tissues.

To further delineate the therapeutic contribution of E2-enhanced ED-ADSCs, we employed a male rat model of CLI as a proof-of-concept system. This design minimized potential confounding effects from endogenous estrogen and allowed evaluation of cell-intrinsic therapeutic effects. In contrast to systemic estrogen administration, in vitro E2 treatment provides a more controlled means of modulating cellular function. It may also reduce potential off-target effects and safety concerns associated with systemic hormone exposure. In this setting, treatment with ED-ADSCs alone resulted in modest and non-significant improvement in blood flow recovery, whereas E2-treated ED-ADSCs showed improved perfusion, particularly at later time points. These findings suggest that E2-associated modulation of endothelial characteristics may contribute to enhanced therapeutic responses in ischemic tissue repair. This strategy may provide a potential approach for modulating cell function without systemic hormone exposure.

Several limitations of the present study should be acknowledged. First, the magnitude of changes observed across molecular, signaling, and functional assays was modest, likely reflecting the elevated baseline under endothelial differentiation conditions. In addition, ADSCs represent a heterogeneous population, with only a small subset of cells exhibiting endothelial-associated characteristics, and the lack of enrichment for EC-like cells may have limited the magnitude of the observed responses.

Methodologically, the Matrigel-based tube formation assay has limited specificity and may not fully reflect in vivo angiogenic potential, and could be complemented by more specific assays, such as fibroblast co-culture systems, in future studies. Similarly, signaling analyses were performed under steady-state differentiation conditions rather than serum-starved acute stimulation, which may limit interpretation of signaling dynamics. Furthermore, gene expression changes detected by qPCR were generally modest and did not consistently reach a two-fold threshold, supporting a modulatory rather than robust transcriptional response. In addition, total eNOS protein levels were not assessed, which may limit interpretation of phosphorylation data.

At the in vivo level, direct assessment of vascularization and detailed histological outcomes (e.g., necrosis or tissue remodeling) were not performed, and cell tracking analyses were primarily qualitative. In addition, the use of a limited marker panel (e.g., CD31) does not fully define endothelial or progenitor cell identity. In addition, sex-specific differences were not assessed, and potential differences between male- and female-derived ADSCs cannot be excluded. Finally, although the observed signaling responses are consistent with ER-mediated mechanisms, the absence of direct ER inhibition or silencing experiments precludes definitive mechanistic conclusions. Collectively, these limitations suggest that the findings should be interpreted as indicative of modest modulation of endothelial-associated characteristics rather than definitive endothelial differentiation or strong therapeutic enhancement.

## 5. Conclusions

The present study suggests that E2 is associated with modulation of endothelial-associated characteristics in adipose-derived stromal cells, accompanied by changes in ER-associated signaling pathways, including PI3K/Akt/eNOS and MAPK cascades, as well as upregulation of pro-angiogenic gene expression. E2 preconditioning was associated with improved blood flow recovery in ischemic limbs in this experimental setting. Collectively, these findings support a modulatory role of estrogen signaling in stem cell-associated vascular repair and suggest that E2 preconditioning may represent a potential approach for enhancing regenerative responses, particularly under estrogen-deficient conditions.

## Data Availability

Data is contained within the article.

## Abbreviations

The following abbreviations are used in this manuscript:

ADSC	Adipose-derived stromal cell
ED	Endothelial differentiation
CLI	Critical limb ischemia
OVX	Ovariectomy
E2	17β-estradiol
